# Vegetable and fruit consumption and cancer of unknown primary risk: results from the Netherlands cohort study on diet and cancer

**DOI:** 10.1186/s12885-022-09502-7

**Published:** 2022-04-13

**Authors:** Karlijn E. P. E. Hermans, Piet A. van den Brandt, Caroline Loef, Rob L. H. Jansen, Leo J. Schouten

**Affiliations:** 1 Department of Epidemiology, GROW School for Oncology and Reproduction, Maastricht University, PO Box 616, 6200 MD Maastricht, the Netherlands; 2 Department of Research, Comprehensive Cancer Organization the Netherlands, Amsterdam, the Netherlands; 3 Department of Internal Medicine, Medical Oncology, Maastricht University Medical Center, Maastricht, the Netherlands

**Keywords:** Cancer of unknown primary (CUP), Vegetable, Fruit, Prospective cohort study

## Abstract

**Background:**

Cancer of Unknown Primary (CUP) is a metastatic cancer for which the primary lesion remains unidentifiable during life and little is also known about the modifiable risk factors that contribute to its development. This study investigates whether vegetables and fruits are associated with CUP risk.

**Methods:**

We used data from the prospective Netherlands Cohort Study on Diet and Cancer which includes 120,852 participants aged between 55 and 69 years in 1986. All participants completed a self-administered questionnaire on cancer risk factors at baseline. Cancer follow-up was established through record linkage to the Netherlands Cancer Registry and the Dutch Pathology Registry. As a result, 867 incident CUP cases and 4005 subcohort members were available for case-cohort analyses after 20.3 years of follow-up. Multivariable adjusted hazard ratios were calculated using proportional hazards models.

**Results:**

We observed no associations between total vegetable and fruit consumption (combined or as separate groups) and CUP risk. However, there appeared to be an inverse association between the consumption of raw leafy vegetables and CUP. With respect to individual vegetable and fruit items, we found neither vegetable nor fruit items to be associated with CUP risk.

**Conclusions:**

Overall, vegetable and fruit intake were not associated with CUP incidence within this cohort.

**Supplementary Information:**

The online version contains supplementary material available at 10.1186/s12885-022-09502-7.

## Background

Cancer of Unknown Primary (CUP) is a metastasised malignancy for which the primary tumor origin remains unidentifiable during life [1, 2]. A historical study has estimated that CUP accounts for 3-5% of all epithelial tumours [3, 4]. In a more recent study, it was observed that CUP incidence has decreased over the last 10-20 years. This decline in CUP incidence was investigated by comparing population-based incidence-rates, and its authors concluded that the decrease could possibly be explained due to advanced imaging and molecular profiling [5]. In the Netherlands, the disease accounted for approximately 1300 incident cases, which represented almost 2% of all new cancer diagnoses in 2018 [6, 7]. The median survival of CUP patients is 1.7 months (2000-2012) [2]. To prevent CUP, it may be beneficial to identify modifiable lifestyle risk factors that have been associated with other cancers. To date, modifiable risk factors that have been associated with CUP are cigarette smoking, and alcohol consumption (dose-response) [8–11]. However, the relationship between diet and CUP has been less studied, especially with respect to plant-based nutrition such as vegetables and fruits.

The World Cancer Research Fund reports that the consumption of vegetables and fruits may reduce cancer risk, although the association may be restricted to specific cancers [12–14]. In addition, they describe that non-starchy vegetables and fruits have been linked to protecting against a number of aerodigestive cancers [12, 13]. Associations between diet and cancer are complex as each bioactive food constituent has the potential to modify aspects of carcinogenesis, either individually or in combination with several micronutrients (alongside quantity, timing, and duration of exposure to those constituents) [12]. Then again, a lower intake of vegetables and fruits (low intake levels of carotenoids, vitamin A, C, E) has been linked to increase levels of oxidative stress and inflammation, alongside genomic instability, reduced apoptosis and increased proliferation [14].

To the best of our knowledge, only one Australian prospective cohort study has investigated the relationship between diet and CUP, in which they did not find any associations between vegetable or fruit consumption and CUP risk [10]. However, it should be noted that the study only examined vegetable and fruit consumption by using the usual number of servings as ≥5 vegetables/day and ≥ 2 fruits/day in relation to CUP. Similarly, it did not investigate specific groups of vegetables and fruits, nor individual vegetable and fruit items. For that reason, we decided to investigate the relationship between vegetable and fruit consumption and CUP risk in greater detail by using combined groups of vegetables and fruits, as well as individual vegetable and fruit items. In addition, we aimed to examine residual confounding by cigarette smoking status on the association between vegetable and fruit consumption and CUP risk, as cigarette smoking has been linked to increase CUP risk.

## Methods

### Study design and population

**Figure 1:**
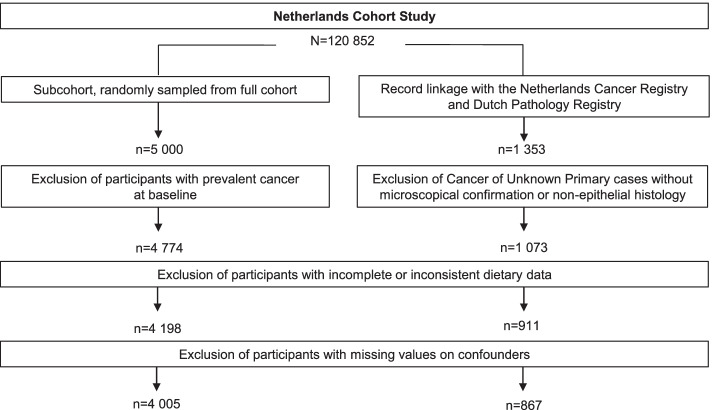
Fig. 1

The prospective Netherlands Cohort Study on Diet and Cancer (NLCS) was started in September 1986 and included 58,279 men and 62,573 women aged between 55 and 69 years. Participants originated from 204 Dutch computerized municipal population registries. Data processing and analysis were based on the case-cohort design for efficiency reasons. Incident cancer cases were derived from the full cohort while the number of person-years at risk was estimated from a subcohort of 5000 participants who were randomly sampled from the full cohort immediately after baseline [15]. The subcohort comprises a group of participants in which CUP cases can occur [16]. The case-cohort design implies that cases can arise both inside and outside the subcohort. The cases in the subcohort are at risk from baseline until cancer incidence, cases outside the subcohort have been assigned a minimal person-time at risk in order to be included in the statistical analysis. Participants who had reported a history of cancer (except for skin cancer) at baseline were excluded from analyses (see Fig. 1).

### Outcome measure

CUP is defined here as a metastasised epithelial malignancy with no identifiable primary tumor origin after cytological and/or histological verification during a patient’s lifetime. This CUP definition only includes epithelial malignancies (ICD-O-3: M-8000 - M-8570) and thus excludes non-epithelial cancers, such as sarcoma, lymphoma, mesothelioma, and melanoma.

### Follow-up

Cancer follow-up was established through annual record linkage with the Netherlands Cancer Registry (NCR) and the Dutch Pathology Registry (PALGA) [17]. Information regarding the site of metastasis was obtained from the NCR, but this data was only partially available and, therefore, supplementary information was retrieved from the pathology excerpts provided by PALGA. These pathology excerpts were also used to determine whether cytological and/or histological confirmed cases had been correctly categorised in the data received from the NCR.

### Questionnaire data

All cohort members completed a self-administered questionnaire, which included detailed questions on dietary habits, lifestyle, and other cancer risk factors. The dietary section was a validated 150-item semi quantitative food-frequency questionnaire (FFQ) that concentrated on the habitual consumption of foods and beverages during the year preceding baseline [18]. The Spearman correlation coefficient was 0.38 for total vegetable consumption and 0.60 for total fruit consumption, compared to the results of the 9 recording days. The relatively low correlation for total vegetable consumption may derive from lack of variation in consumption and possibly due to imprecise estimation of the portion size [18, 19]. Participants were asked to indicate how often they consumed vegetables (15 cooked vegetables, 4 raw vegetables), both in summer and in winter. They were able to choose from one out of six categories: never or less than once a month, 1 time per month, 2 to 3 times per month, 1 time per week, 2 times per week, or 3 to 7 times per week. Usual serving sizes were asked for string beans and cooked endive only; the mean of these values served as an indicator for serving sizes of all cooked vegetables. Participants who did not report their usual serving sizes were assigned a default value. If participants reported only one serving size, then the individual serving size was derived using a conversion factor. Both the default value and the conversion factor were derived from a pilot study [20]. Tomato and sweet pepper consumption were asked to be reported in frequency per week and per month, respectively, both in summer and in winter. Participants were asked to indicate how often they consumed fruit by choosing from one out of seven categories: never or less than once a month, 1 time per month, 2 to 3 times per month, 1 time per week, 2 to 3 times per week, 4 to 5 times per week, or 6 to 7 times per week. For all the fruits of interest, participants were able to indicate the amount of each fruit that was consumed. Frequencies and amounts were converted to grams per day. For both vegetable and fruit consumption, dietary data measured in summer and winter were merged and averaged into specific intake variables for analyses purposes. The questionnaire was also used to measure exposure to tobacco smoking. Tobacco smoking was addressed through questions on baseline smoking status, and the ages at first exposure and last (if stopped) exposure to smoking. Questions were also asked about smoking frequency and smoking duration (excluding stopping periods), for cigarette, cigar, and pipe smokers. Participants who indicated that they had never smoked cigarettes were considered never smokers.

### Statistical methods

Person-years at risk were calculated from baseline (17 September 1986) until CUP diagnosis, death, emigration, loss to follow-up, or end of follow-up (31 December 2006), whichever occurred first. Patient characteristics were presented for CUP cases and stratified for histological and cytological confirmation. General characteristics were presented for subcohort members and CUP cases with frequencies (percentages) for categorical variables, and means including standard deviations for continuous variables.

Based on the distribution of the subcohort, participants were compared using quartiles (Q) of vegetable, legume, and fruit consumption. For continuous analyses, increments of 25 g per day were used. The composition of the vegetable, legume, and fruit groups that were studied within the NLCS are described in Table 1.

**Table 1:** Composition of vegetable and fruit groups, based on vegetable and fruit items that were asked in the food-frequency questionnaire in the Netherlands Cohort Study

Food group	Composition
Total vegetables	Cooked vegetables plus raw vegetables
Cooked vegetables	Beetroot, broad beans, Brussels sprouts, cauliflower, cabbage (white/green), cooked carrots, cooked endive, kale, leek, mushrooms, onions, rhubarb, sauerkraut, spinach, string beans, sweet peppers and other cooked vegetables originating from an open-ended question on frequently consumed items not listed in the questionnaire
Raw vegetables	Gherkins, lettuce, raw carrots, raw endive, tomatoes and other raw vegetables from an open-ended question on frequently consumed items not listed in the questionnaire
Brassica vegetables	Brussels sprouts, cabbage (white/green), cauliflower and kale
Leafy vegetables, cooked	Cooked endive and spinach
Leafy vegetables, raw	Lettuce and raw endive
Legumes	Broad beans, dried pulses and string beans
Allium vegetables	Leek and onions
Total fruits	Apples/pears, bananas, grapefruits and fresh grapefruit juice, grapes, mandarins, oranges and fresh orange juice, raisins/other dried fruit, strawberries and other fruits originating from an open-ended question on frequently consumed items not listed in the questionnaire
Citrus fruits	Fresh lemon juice, grapefruits and fresh grapefruit juice, mandarins, oranges and fresh orange juice

Vegetable and fruit consumption were mutually adjusted in the analyses, which means that vegetable consumption was additionally adjusted for fruit consumption, whereas fruit consumption was additionally adjusted for vegetable consumption. Legume consumption was additionally adjusted for vegetable and fruit intake. The predefined confounders included: age at baseline (years, continuous); sex (male/female); current cigarette smoking status (never/ever); cigarette smoking frequency (number of cigarettes smoked per day); and cigarette smoking duration (number of years smoking). We included the smoking variables as predefined confounders, as they have been linked to increased CUP risk [8–11]. Additionally, smokers have been observed to consume lower amounts of vegetables and fruits in comparison to non-smokers [21]. The potential confounders included: alcohol consumption (ethanol intake per day); body mass index (BMI) at baseline (kg/m^2^); non-occupational physical activity (< 30 min/day, 30-60 min/day, 60-90 min/day and > 90 min/day); socio-economic status (highest level of education); diabetes (yes/no); and history of cancer in a first-degree relative (yes/no). Variables were considered a confounder if they changed the HR by > 10%. Accordingly, none of the potential confounders were included in the final model.

Cox proportional hazards models were used to estimate age- and sex-adjusted, and multivariable adjust hazard ratios (HRs) with 95% confidence intervals (CIs). Time since baseline (1986) was used for the time axis. Standard errors were calculated using the robust Huber-White sandwich estimator to account for additional variance introduced by sampling from the full cohort [22]. The proportional hazards assumption was tested using the scaled Schoenfeld residuals [23]. In cases where the assumption had been violated, a time-varying coefficient for that variable was added to the model where appropriate. Ordinal exposure variables were fitted as continuous variables in trend analyses. Wald tests and cross-product terms were used to evaluate potential multiplicative interaction between total vegetable and fruit consumption (combined and individually), with sex, and CUP risk, and between total vegetable and fruit consumption (combined and individually), cigarette smoking frequency, and CUP risk. Analyses were conducted using Stata version 15. *P* values were considered statistically significant if *p* < 0.05.

We performed three sensitivity analyses. The first sensitivity analysis was restricted to histologically verified CUP cases alone. For this analysis, patients who received a cytological verification alone were excluded*.* Patients who were histologically verified are more likely to have undergone extensive diagnostic investigation(s) to rule out the primary tumour origin. For those patients who received cytological verification alone, other factors may have played a role in the decision to refrain from further diagnostic investigation, such as age, comorbidities, performance status, localisation of the metastasis, and the patient’s decision. The second sensitivity analysis was performed after the first 2 years of follow-up had been excluded so as to check for potential reverse causality bias as a result of preclinical cancer at baseline. To assess whether associations differed over time, we conducted a third analysis in which we compared the first 10 years of follow-up (< 1996) to the last 10 years of follow-up (≥1996).

## Results

After 20.3 years of follow-up (17 September 1986 until 31 December 2006), data was available for a total of 1353 potential CUP cases and 4774 participants of the subcohort. After excluding CUP cases with neither microscopical confirmation or non-epithelial histology, a total of 1073 CUP cases remained. Participants with incomplete or inconsistent dietary data were excluded from analyses. This resulted in 867 available CUP cases and 4005 subcohort members with complete and consistent dietary data. In general, when comparing differences between CUP cases and subcohort members, we observed that CUP cases consumed lower amounts of vegetables (mean values 185.8 versus 189.0 g per day, respectively) (see Table 2). Male CUP cases in particular consumed lower amounts of vegetables (mean values 182.3 versus 187.0 g per day, respectively), while female CUP cases consumed a more similar amount of vegetables (mean values 191.6 versus 190.9 g per day, respectively). We also observed that CUP cases consumed lower amounts of fruits (mean values 164.7 versus 175.5 g per day, respectively).

**Table 2:** Characteristics of Cancer of Unknown Primary cases and subcohort members in the Netherlands Cohort Study at baseline

	Subcohort members				Cancer of Unknown Primary cases			
	(***n*** = 4005)				(***n*** = 867)			
Characteristic	n	(%)	mean	SD	n	(%)	mean	SD
**Age at baseline (years)**								
55-59	1550	38.7			265	30.6		
60-64	1389	34.7			340	39.2		
65-69	1066	26.6			262	30.2		
**Sex**								
Men	1941	48.5			537	61.9		
Women	2064	51.5			330	38.1		
**Total vegetable and fruit consumption (g/day)**			364.5	152.4			350.5	145.5
Men			342.4	149.6			329.5	142.4
Women			385.2	152.1			384.7	144.3
**Total vegetable consumption (g/day)**			189.0	75.5			185.8	74.2
Men			187.0	76.0			182.3	75.1
Women			190.9	75.1			191.6	72.5
**Total fruit consumption (g/day)**			175.5	118.2			164.7	113.8
Men			155.4	114.5			147.3	110.4
Women			194.4	118.5			193.1	113.7
**Ethanol intake (grams/day)** ^**a**^								
Abstainers	920	23.6			155	18.2		
< 5	1105	28.4			220	25.9		
5- < 15	896	23.0			196	23.0		
15- < 30	623	16.0			136	16.0		
≥ 30	354	9.1			144	16.9		
**Cigarette smoking status**								
Never smokers	1500	37.5			252	29.1		
Ex smokers	1439	35.9			304	35.1		
Current smokers	1066	26.6			311	35.9		
**Frequency of cigarette smoking (N/day)** ^**a**^			15.7	10.0			17.8	10.1
**Duration of cigarette smoking (years)** ^**a**^			31.8	12.1			35.3	11.7
**Body Mass Index at baseline (kg/m**^**b**^**)**			25.0	3.1			24.9	3.0
**Non-occupational physical activity (min/day)**								
≤ 30	838	21.2			181	21.2		
> 30-60	1240	31.4			261	30.6		
> 60-90	834	21.1			154	18.1		
> 90	1043	26.4			257	30.1		
**Level of education (years of education)**								
Primary	1137	28.9			229	26.6		
Lower vocational	857	21.5			172	20.0		
Secondary and medium vocational	1423	35.7			328	38.1		
University and higher vocational	566	14.2			131	15.2		
**Diabetes**								
Yes	138	3.5			31	3.6		
**First grade family history of cancer** ^**c**^								
Yes	1836	45.8			422	48.7		

^a^In consumers only

^**b**^In users only

^**c**^First degree relative with cancer

Results from the age- and sex-adjusted analyses were comparable to the results of the multivariable adjusted analyses. Therefore, we only discuss the multivariable adjusted results. We observed no association between total vegetable and fruit consumption (HR for Q4 vs. Q1: 0.98, 95% CI: 0.92-1.05, *P*_trend_ = 0.63) and CUP risk (see Table 3). In addition, when mutually adjusted, we found no association between total vegetables (HR for Q4 vs. Q1: 0.87, 95% CI: 0.69-1.09, *P*_trend_ = 0.38) or total fruits (HR for Q4 vs. Q1: 0.94, 95% CI: 0.75-1.17, *P*_trend_ = 0.56) and CUP risk. Furthermore, we found no associations between the following vegetable groups: cooked vegetables (HR for Q4 vs. Q1: 1.06, 95% CI: 0.82-1.38, *P*_trend_ = 0.71), raw vegetables (HR for Q4 vs. Q1: 0.96, 95% CI: 0.75-1.22, *P*_trend_ = 0.94), legumes (HR for Q4 vs. Q1: 1.21, 95% CI: 0.97-1.52, *P*_trend_ = 0.14), brassica vegetables (HR for Q4 vs. Q1: 1.01, 95% CI: 0.81-1.27, *P*_trend_ = 0.92), allium vegetables (HR for Q4 vs. Q1: 1.14, 95% CI: 0.91-1.42, *P*_trend_ = 0.48), cooked leafy vegetables (HR for Q4 vs. Q1: 0.92, 95% CI: 0.74-1.15, *P*_trend_ = 0.68), or the fruit group: citrus fruits (HR for Q4 vs. Q1: 1.15, 95% CI: 0.93-1.42, *P*_trend_ = 0.37) and CUP risk. However, we observed a statistically significant trend between the consumption of raw leafy vegetables and a decreased CUP risk (HR for Q4 vs. Q1: 0.82, 95% CI: 0.64-1.03, *P*_trend_ = 0.03). With respect to individual vegetable and fruit items, which were mutually adjusted, we found no association between the individual vegetable items or the individual fruit items and the development of CUP (see Table 4).

**Table 3:** Hazard ratios and 95% confidence intervals for vegetable and fruit consumption and Cancer of Unknown Primary risk in the Netherlands Cohort Study

	Categorical median (grams per day)		Subcohort members	Cancer of Unknown Primary cases						
			(***n*** = 4005)	(***n*** = 867)						
			Person time at risk (years)	Cases	Age- and sex- adjusted			Multivariable adjusted ^**a**^		
	Men	Women		n	HR	95% CI		HR	95% CI	
**Total vegetables and fruits**										
Q1	188	226	16,680	224	1	Reference		1	Reference	
Q2	282	323	16,957	224	0.96	(0.78-1.19)		1.02	(0.83-1.27)	
Q3	363	411	16,989	209	0.90	(0.72-1.11)		0.96	(0.78-1.19)	
Q4	496	552	17,184	210	0.87	(0.70-1.07)		0.97	(0.78-1.20)	
*p* for trend^b^					0.14			0.63		
Continuous, 25 g per day increments			67,810	867	0.95	(0.89-1.02)		0.98	(0.92-1.05)	
**Total vegetables** ^**c**^										
Q1	109	124	16,600	228	1	Reference		1	Reference	
Q2	156	160	17,022	211	0.91	(0.74-1.13)		0.94	(0.76-1.17)	
Q3	199	202	17,172	233	0.99	(0.80-1.22)		1.04	(0.84-1.28)	
Q4	271	277	17,016	195	0.84	(0.68-1.04)		0.87	(0.69-1.09)	
*p* for trend^b^					0.21			0.38		
Continuous, 25 g per day increments			67,810	867	0.96	(0.90-1.02)		0.97	(0.90-1.04)	
**Cooked vegetables** ^**d**^										
Q1	85	86	16,707	223	1	Reference		1	Reference	
Q2	125	124	16,976	216	0.96	(0.77-1.18)		1.00	(0.80-1.24)	
Q3	160	159	17,320	213	0.93	(0.75-1.15)		0.99	(0.79-1.24)	
Q4	220	216	16,806	215	0.96	(0.78-1.19)		1.06	(0.82-1.38)	
*p* for trend^b^					0.69			0.71		
Continuous, 25 g per day increments			67,810	867	0.99	(0.92-1.06)		1.02	(0.94-1.10)	
**Raw vegetables** ^**d**^										
Q1	8	11	16,680	221	1	Reference		1	Reference	
Q2	24	29	16,982	217	0.95	(0.77-1.17)		1.04	(0.84-1.29)	
Q3	39	45	17,014	235	1.02	(0.83-1.25)		1.12	(0.90-1.39)	
Q4	67	72	17,134	194	0.85	(0.68-1.05)		0.96	(0.75-1.22)	
*p* for trend^b^					0.23			0.94		
Continuous, 25 g per day increments			67,810	867	0.96	(0.90-1.03)		0.99	(0.93-1.07)	
**Legumes** ^**d**^										
Q1	13	11	16,934	203	1	Reference		1	Reference	
Q2	24	21	17,036	217	1.09	(0.88-1.35)		1.11	(0.90-1.38)	
Q3	36	32	17,055	214	1.07	(0.86-1.32)		1.08	(0.87-1.35)	
Q4	57	52	16,784	233	1.20	(0.97-1.48)		1.21	(0.97-1.52)	
*p* for trend^b^					0.13			0.14		
Continuous, 25 g per day increments			67,810	867	1.05	(0.98-1.13)		1.06	(0.98-1.14)	
**Brassica vegetables** ^**d**^										
Q1	12	12	16,718	228	1	Reference		1	Reference	
Q2	24	23	17,043	214	0.94	(0.76-1.16)		0.95	(0.77-1.18)	
Q3	35	33	17,162	205	0.88	(0.71-1.09)		0.89	(0.71-1.11)	
Q4	54	53	16,888	220	0.97	(0.79-1.20)		1.01	(0.81-1.27)	
*p* for trend^b^					0.68			0.92		
Continuous, 25 g per day increments			67,810	867	0.99	(0.92-1.06)		0.99	(0.92-1.07)	
**Allium vegetables** ^**d**^										
Q1	6	4	18,455	240	1	Reference		1	Reference	
Q2	19	20	15,155	199	1.04	(0.84-1.28)		1.07	(0.86-1.33)	
Q3	31	33	17,224	195	0.91	(0.74-1.12)		0.94	(0.75-1.16)	
Q4	55	55	16,975	233	1.06	(0.87-1.31)		1.14	(0.91-1.42)	
*p* for trend^b^					0.84			0.48		
Continuous, 25 g per day increments			67,810	867	1.01	(0.94-1.08)		1.03	(0.96-1.10)	
**Leafy vegetables, cooked** ^**d**^										
Q1	5	5	16,925	232	1	Reference		1	Reference	
Q2	15	15	16,985	211	0.89	(0.72-1.09)		0.89	(0.72-1.11)	
Q3	24	24	17,051	218	0.95	(0.77-1.17)		0.99	(0.80-1.22)	
Q4	39	38	16,849	206	0.90	(0.72-1.10)		0.92	(0.74-1.15)	
*p* for trend^b^					0.40			0.68		
Continuous, 25 g per day increments			67,810	867	0.97	(0.91-1.04)		0.99	(0.92-1.06)	
**Leafy vegetables, raw** ^**d**^										
Q1	1	1	12,911	197	1	Reference		1	Reference	
Q2	4	4	15,347	217	0.96	(0.77-1.20)		0.98	(0.78-1.22)	
Q3	9	9	21,890	252	0.77	(0.62-0.95)		0.80	(0.64-0.99)	
Q4	20	20	17,661	201	0.77	(0.61-0.96)		0.82	(0.64-1.03)	
*p* for trend^b^					0.004			0.03		
Continuous, 25 g per day increments			67,810	867	0.90	(0.84-0.97)		0.92	(0.85-0.99)	
**Total fruits** ^**e**^										
Q1	41	74	16,675	236	1	Reference		1	Reference	
Q2	109	144	16,980	216	0.88	(0.71-1.09)		0.94	(0.76-1.16)	
Q3	165	210	17,040	205	0.81	(0.66-1.00)		0.92	(0.74-1.15)	
Q4	270	326	17,115	210	0.82	(0.66-1.01)		0.94	(0.75-1.17)	
*p* for trend^b^					0.05			0.56		
Continuous, 25 g per day increments			67,810	867	0.93	(0.87-0.99)		0.98	(0.91-1.05)	
**Citrus fruits** ^**e**^										
Q1	0	6	16,947	222	1	Reference		1	Reference	
Q2	15	36	17,118	213	0.93	(0.75-1.15)		0.98	(0.79-1.21)	
Q3	52	83	16,845	180	0.77	(0.62-0.96)		0.85	(0.68-1.06)	
Q4	115	167	16,900	252	1.07	(0.87-1.31)		1.15	(0.93-1.42)	
*p* for trend^b^					0.84			0.37		
Continuous, 25 g per day increments			67,810	867	1.01	(0.94-1.08)		1.03	(0.96-1.11)	

^a^Analyses were adjusted for age at baseline (years), sex, cigarette smoking status (never/ever), frequency (continuous; centered), and duration (continuous; centered). Additionally adjusted for cigarette smoking status (never/ever), and duration (continuous; centered) as time-varying covariates

^b^Tests for dose-response trends were assessed by fitting ordinal variables as continuous terms in the Cox proportional hazards model

^c^Additionally adjusted for total fruit consumption (grams per day; continuous)

^d^Additionally adjusted for total vegetable and fruit consumption (grams per day; continuous)

^e^Additionally adjusted for total vegetable consumption (grams per day; continuous)

**Table 4:** Hazard ratios and 95% confidence intervals for individual vegetable and fruit items and Cancer of Unknown Primary risk in the Netherlands Cohort Study ^a^

	Follow-up time (years)	Cancer of Unknown Primary cases (***n*** = 867)			
		Age- and sex- adjusted		Multivariable adjusted ^**b**^	
		HR	95% CI	HR	95% CI
**Vegetable item (25 g per day increments)**					
String/French beans	20.3	1.02	(0.90-1.15)	1.01	(0.89-1.15)
Cauliflower	20.3	0.95	(0.80-1.14)	0.95	(0.79-1.15)
Lettuce	20.3	0.75	(0.57-1.01)	0.83	(0.62-1.13)
Carrots, cooked^c^	0-10	0.95	(0.68-1.31)	1.03	(0.75-1.41)
	10-20.3	0.73	(0.55-0.97)	0.78	(0.59-1.03)
Endive, cooked^c^	0-10	0.99	(0.75-1.31)	1.01	(0.76-1.33)
	10-20.3	0.83	(0.66-1.06)	0.85	(0.67-1.08)
Brussels sprouts	20.3	1.04	(0.81-1.35)	1.06	(0.81-1.37)
Sauerkraut	20.3	1.07	(0.75-1.52)	1.12	(0.78-1.62)
Tomatoes	20.3	0.96	(0.87-1.06)	0.98	(0.89-1.08)
Onion	20.3	0.99	(0.90-1.10)	1.02	(0.91-1.13)
Spinach	20.3	0.99	(0.80-1.22)	1.02	(0.82-1.27)
Beetroot^c^	0-10	0.91	(0.64-1.28)	0.99	(0.69-1.41)
	10-20.3	0.60	(0.42-0.85)	0.64	(0.44-0.92)
Kale	20.3	0.86	(0.49-1.52)	0.93	(0.53-1.63)
**Fruit item (25 g per day increments)**					
Apples and pears^c^	0-10	0.95	(0.91-0.99)	0.97	(0.94-1.01)
	10-20.3	0.98	(0.95-1.01)	0.99	(0.96-1.03)
Strawberries	20.3	0.99	(0.77-1.27)	1.06	(0.83-1.36)
Oranges and fresh orange juice	20.3	1.01	(0.97-1.04)	1.03	(0.99-1.07)

^a^The total person time at risk in the subcohort was 67,810 years

^b^Analyses were adjusted for age at baseline (years), sex, cigarette smoking status (never/ever), frequency (continuous; centered), duration (continuous; centered), and total vegetable and fruit consumption (grams per day; continuous). All items were assessed while additionally using cigarette smoking status (never/ever), and duration (continuous; centered) as time-varying covariates

^c^The proportional hazards assumption was violated for the exposure variable in this analysis, consequently these associations were splitted based on follow-up time

No multiplicative interactions were observed between sex and the association between total vegetable and fruit consumption (combined), vegetable consumption, or fruit consumption, in relation to CUP risk (*P*_interaction_ = 0.20, 0.17, and 0.46, respectively). However, we did observe multiplicative interactions between vegetables and fruits (combined), and fruit consumption and smoking status in relation to CUP risk (*P*_interaction_ = 0.03, 0.02, respectively), but not between vegetable consumption and smoking status in relation to CUP risk (*P*_interaction_ = 0.67). Furthermore, the potential for residual confounding was evaluated based on cigarette smoking status and the relationship between vegetable and fruit consumption and CUP risk (see Table 5). In current smokers, the association of vegetables and fruits with CUP risk was inverse, although not statistically significant (per 25 g per day increment HR: 0.89, 95% CI: 0.79-1.00, *P*_trend_ = 0.06). In never and ex-smokers, vegetable and fruit consumption was not associated with CUP risk. Furthermore, current smokers with the highest fruit intake compared to the lowest fruit intake appeared to have a reduced CUP risk (HR for Q4 vs. Q1: 0.65, 95% CI: 0.43-0.99, although the *P*_trend_ = 0.16 was not statistically significant).

**Table 5:** Hazard ratios and 95% confidence intervals for vegetable and fruit consumption and Cancer of Unknown Primary risk in the Netherlands Cohort Study, stratified for cigarette smoking status

	Never smokers					Ex smokers					Current smokers					
	Subcohort members	Cancer of Unknown Primary cases				Subcohort members	Cancer of Unknown Primary cases				Subcohort members	Cancer of Unknown Primary cases				
	(***n*** = 1500)	(***n*** = 252)				(***n*** = 1439)	(***n*** = 304)				(***n*** = 1066)	(***n*** = 311)				
	Person time at risk (years)	Cases	Age- and sex-adjusted ^**a**^			Person time at risk (years)	Cases	Age- and sex-adjusted ^**a**^			Person time at risk (years)	Cases	Age- and sex-adjusted ^**a**^			
		n	HR	95% CI			n	HR	95% CI			n	HR	95% CI		***p*** for interaction ^**b**^
**Total vegetables and fruits**																0.032
Q1	6185	53	1	Reference		5006	59	1	Reference		5489	112	1	Reference		
Q2	6470	66	1.17	(0.79-1.74)		6227	76	1.05	(0.71-1.54)		4260	82	0.95	(0.68-1.34)		
Q3	6846	70	1.19	(0.81-1.76)		6201	65	0.95	(0.64-1.40)		3941	74	0.86	(0.61-1.22)		
Q4	7435	63	0.92	(0.62-1.38)		6677	104	1.35	(0.94-1.94)		3072	43	0.67	(0.45-1.01)		
*p* for trend^c^			0.66					0.13					0.06			
Continuous, 25 g per day increments	26,935	252	0.97	(0.87-1.09)		24,112	304	1.10	(0.97-1.24)		16,763	311	0.89	(0.79-1.00)		
**Total vegetables**																0.673
Q1	7081	63	1	Reference		5102	69	1	Reference		4417	96	1	Reference		
Q2	6800	64	1.10	(0.75-1.60)		6049	72	0.88	(0.61-1.29)		4172	75	0.92	(0.64-1.32)		
Q3	6751	71	1.25	(0.86-1.81)		6294	88	1.08	(0.75-1.55)		4127	74	0.88	(0.61-1.26)		
Q4	6303	54	1.04	(0.69-1.56)		6666	75	0.79	(0.54-1.16)		4046	66	0.81	(0.55-1.19)		
*p* for trend^c^			0.65					0.38					0.27			
Continuous, 25 g per day increments	26,935	252	1.03	(0.91-1.16)		24,112	304	0.95	(0.85-1.07)		16,763	311	0.93	(0.83-1.05)		
**Total fruits**																0.019
Q1	5650	53	1	Reference		5144	67	1	Reference		5881	116	1	Reference		
Q2	6415	68	1.15	(0.77-1.70)		5980	65	0.83	(0.56-1.22)		4585	83	0.91	(0.65-1.27)		
Q3	7150	59	0.84	(0.56-1.27)		6616	74	0.91	(0.62-1.32)		3274	72	1.10	(0.76-1.56)		
Q4	7720	72	0.93	(0.62-1.38)		6372	98	1.22	(0.84-1.77)		3023	40	0.65	(0.43-0.99)		
*p* for trend^c^			0.39					0.20					0.16			
Continuous, 25 g per day increments	26,935	252	0.95	(0.83-1.07)		24,112	304	1.09	(0.96-1.23)		16,763	311	0.92	(0.82-1.03)		

^a^Analyses were adjusted for age at baseline (years) and sex

^b^Interactions were calculated with respect to smoking status in relation to the vegetable/fruit variable of interest and CUP risk

^c^Tests for dose-response trends were assessed by fitting ordinal variables as continuous terms in the Cox proportional hazards model

Results from all three sensitivity analyses, when restricted to histologically verified CUP cases alone (*n* = 614), after excluding the first 2 years of follow-up, and when comparing the first 10 years of follow-up (< 1996) to the last 10 years of follow-up (≥1996), did not differ substantially from the findings of the overall analyses (see Supplementary Tables 1-6).

## Discussion

We have presented here a detailed investigation of the relationship between vegetable and fruit consumption and the development of CUP, which we accomplished by assessing combined groups of vegetables and fruits as well as individual vegetable and fruit items. Our results demonstrate that consuming vegetables and fruits is generally unrelated to CUP incidence within this cohort; however, the consumption of raw leafy vegetables did appear to be associated with a decreased CUP risk. We found no multiplicative interaction between sex in relation to the association between total vegetable and fruit consumption and CUP risk. Yet, we did observe multiplicative interactions between total vegetables and fruits (combined), and fruit consumption and smoking status in relation to CUP risk, but not between vegetable consumption and smoking status in relation to CUP risk.

The Australian cohort study, mentioned in the introduction, investigated the relationship between consuming vegetables and fruits and the risk of developing CUP by comparing 327 incident CUP cases to two randomly selected sets of controls (3:1) using incidence density sampling with replacement [10]. It found no relation by assessing plant-based food consumption and the usual number of servings as ≥5 vegetables/day and ≥ 2 fruits/day, compared to consuming < 5 vegetables/day and < 2 fruits/day [10]. Although the categories differ between the Australian study and those of the NLCS, the respective findings are comparable. Moreover, having analysed combined groups of vegetables and fruits as well as individual vegetable and fruit items in greater detail, we conclude that there is no association between vegetable and fruit consumption and CUP risk. We did, however, observe an inverse association between the consumption of raw leafy vegetables and CUP risk, but this might be a chance finding due to multiple comparisons. As described elsewhere, vegetable and fruit consumption have been associated with a protective effect against cancer, but the association may be restricted to specific cancers [12]. Nonetheless, it should be acknowledged that CUP constitutes a group of heterogeneous metastatic cancers, therefore, specific effects from vegetables and/or fruits could be masked.

In an additional analysis, residual confounding by cigarette smoking status was evaluated for its possible influence on the association between vegetable and fruit consumption and CUP risk. We observed no associations for never or ex-smokers who consumed vegetables and fruits in relation to CUP risk, while current smokers appeared to have a decreased CUP risk, although not statistically significant. This effect may derive from residual confounding by smoking. Our finding is in line with the limited-suggestive evidence by the World Cancer Research Fund that describes the consumption of non-starchy vegetables and fruit to be linked to reduced lung cancer risk in people who smoke or used to smoke tobacco [13].

### Strengths and limitations

The strengths of this study are its prospective cohort design, its large cohort population including 120,852 participants, its large number of 867 incident CUP cases, and its ability to correct for multiple and detailed confounders in the analyses. Data on incident CUP cases were provided by the NCR and included information from both pathology reports and clinical reports [24]. Pathology excerpts were available to confirm whether the cytological and/or histological confirmed cases had been correctly categorised in the data received from the NCR. Cancer follow-up through record linkage with the NCR and PALGA was at least 96% complete, thereby minimizing selection bias [25]. Cases were registered by trained NCR registry clerks who had access to the medical files and who entered data by applying uniform coding rules. It should, however, be acknowledged that we utilised a CUP definition that may differ from that used in other countries, as the criteria for defining ‘CUP’ are heterogeneous. Another possible limitation is that exposure data were only measured once at baseline in 1986. Vegetable and fruit consumption (both in summer and in winter) were, however, extensively addressed in the FFQ, and we expect that participants in the studied age group (55-69) had stable dietary habits at baseline. The reproducibility of the FFQ as well as the stability of dietary habits as estimated by the test-retest r, was on average 0.07 for nutrients over a time period of 5 years [26]. Nonetheless, it is possible that participants subsequently changed their dietary habits. If they did change their habits, that may have resulted in bias due to misclassification and may have led to underestimation of the effect of vegetable and fruit consumption on CUP risk. We do expect this bias to be non-differential between CUP cases and subcohort members. Unfortunately, we do not have data to check which diagnostic methods were used to identify the primary tumor origin. Nevertheless, if we restrict our analysis to histologically verified CUP cases alone, for whom extended diagnostic methods are more likely, we find that the results do not differ greatly from the overall multivariable analyses. Accordingly, we can assume that the findings from the overall multivariable analyses are representative of CUP cases with or without an extensive diagnostic work-up. We were unable to conduct subgroup analyses based on histopathological findings as precision medicine was not yet available at the time of the follow-up of our study. Studies with more recent data on CUP cases would therefore be encouraged to conduct such analyses.

## Conclusions

In our study, we observed no associations between total vegetable and fruit consumption, total vegetables, cooked vegetables, raw vegetables, legumes, brassica vegetables, allium vegetables, cooked leafy vegetables, total fruits, citrus fruits, and the development of CUP. However, the consumption of raw leafy vegetables appeared to decrease risk of the malignancy. With respect to individual vegetable and fruit items, neither vegetable nor fruit items were found to be associated with CUP risk. We thus conclude that consuming vegetables and fruits is unrelated to CUP incidence within this cohort.

## Supplementary Information

**Additional file 1.**

## Data Availability

The datasets generated and/or analysed during the current study are not publicly available because the informed consent does not allow for that. However, anonymous data that are minimally required to replicate the outcomes of the study will be made available upon reasonable request and approval by the institutional review boards.

## Abbreviations

CI: Confidence interval
CUP: Cancer of Unknown Primary
HR: Hazard ratio
NCR: Netherlands Cancer Registry
NLCS: Netherlands Cohort Study on Diet and Cancer
PALGA: Dutch Pathology Registry
